# Cadmium-Free Quantum Dots as Fluorescent Labels for Exosomes

**DOI:** 10.3390/s18103308

**Published:** 2018-10-02

**Authors:** Garima Dobhal, Deanna Ayupova, Geoffry Laufersky, Zeineb Ayed, Thomas Nann, Renee V. Goreham

**Affiliations:** 1 The MacDiarmid Institute, Wellington 6012, New Zealand; garima.dobhal@vuw.ac.nz (G.D.); deanna.ayupova@vuw.ac.nz (D.A.); geoffry.laufersky@vuw.ac.nz (G.L.); zeineb.ayed@vuw.ac.nz (Z.A.); thomas.nann@vuw.ac.nz (T.N.); 2 School of Chemical Physical Sciences, Victoria University of Wellington, Kelburn, Wellington 6012, New Zealand

**Keywords:** quantum yields, bioimaging, extracellular vesicles, antibodies

## Abstract

Quantum dots are attractive alternatives to organic fluorophores for the purposes of fluorescent labeling and the detection of biomarkers. They can also be made to specifically target a protein of interest by conjugating biomolecules, such as antibodies. However, the majority of the fluorescent labeling using quantum dots is done using toxic materials such as cadmium or lead due to the well-established synthetic processes for these quantum dots. Here, we demonstrate the use of indium phosphide quantum dots with a zinc sulfide shell for the purposes of labeling and the detection of exosomes derived from the THP-1 cell line (monocyte cell line). Exosomes are nano-sized vesicles that have the potential to be used as biomarkers due to their involvement in complex cell processes. However, the lack of standardized methodology around the detection and analysis of exosomes has made it difficult to detect these membrane-containing vesicles. We targeted a protein that is known to exist on the surface of the exosomes (CD63) using a CD63 antibody. The antibody was conjugated to the quantum dots that were first made water-soluble using a ligand-exchange method. The conjugation was done using carbodiimide coupling, and was confirmed using a range of different methods such as dynamic light scattering, surface plasmon resonance, fluorescent microscopy, and Fourier transform infrared spectroscopy. The conjugation of the quantum dot antibody to the exosomes was further confirmed using similar methods. This demonstrates the potential for the use of a non-toxic conjugate to target nano-sized biomarkers that could be further used for the detection of different diseases.

## 1. Introduction

Quantum dots (QDs) are semiconducting nanocrystals that have intrinsic and stable optical properties making them ideal for therapeutic applications. These properties include high quantum yields, high extinction coefficients, and increased stability, which makes them desirable for bioimaging and detection in biomedical applications. Also, compared to alternatives, such as organic fluorophores and dye molecules [1], they can be used for real-time bioimaging. Unlike conventional molecular probes and dyes, antibody-conjugated QDs have great potential for real-time imaging, allowing for advanced imaging with improved sensitivity and resolution [2,3]. Several studies have revealed the advantages of QDs over fluorescent dyes. For example, tumor growth in mice was successfully imaged using QD antibodies (QD Abs), which clearly demonstrated their high stability in vivo and resistance to photobleaching [4]. With that said, commercially available QDs are generally made from cadmium or lead-based nanocrystals, which are known to be highly toxic. A safer alternative are indium-based QDs, as the In(III) ions have a lower intrinsic toxicity compared to the Cd(II) ions [5]. In addition, QDs can be conjugated to a targeting moiety, such as an antibody, which can then specifically label a target, such as cell-derived exosomes, for example.

Exosomes are nano-sized mammalian cell-derived vesicles that have generated interest due to their applications in drug delivery and therapeutics [6]. They are a type of extracellular vesicle that contains information in the form of DNA, RNA, and protein that is unique to the cell from which they originate [6]. Due to their known involvement in pathological conditions such as cancer [7], neurodegenerative diseases, obesity, and pregnancy complications, they have the potential to be used as biomarkers for the detection of diseases [6]. Exosomes are known to contain a variety of proteins in their membrane and the most general class of proteins that is used for targeting all of the exosomes are the tetraspanins (CD9, CD63, CD81) [8,9]. CD63, for example, is consistently used as a protein marker for exosomes [10].

We report the synthesis and conjugation of InP/ZnS QDs with an antibody (Anti-CD63) to specifically target exosomes. First, InP/ZnS QDs with oleylamine ligands were synthesized using a published protocol, after which the QDs were ligand exchanged with a thiolated ligand such as mercaptosuccinic acid (MSA) to render them water-soluble. The carboxyl functional group on MSA and the amino groups present in the antibody formed a covalent bond via carbodiimide chemistry. Anti-CD63 specifically targets the CD63 protein found in the membrane of all exosomes.

An issue with using exosomes as biomarkers is the problems around the consistency of analysis and detection of exosomes. Some common methods for the detection of exosomes is through the use of nanoparticle tracking analysis, flow cytometry, and dynamic light scattering (DLS) [11,12]. However, each of these methods cannot be used in isolation due to their individual limitations. Most of these limitations are based around the size of the particles. For example, the commonly used flow cytometers cannot detect particles under 300 nm. Nanoparticle tracking analysis cannot detect particles under 10 nm, and polydisperse samples, such as a sample with exosomes, can give unreliable results related to both the size and concentration of nanoparticles [11,13]. Transmission electron microscopy (TEM) is one reliable way to analyze a population of exosomes, but it needs expertise around the use of instrumentation.

The QDs that were functionalized with an antibody that is specific for the exosomes would therefore make it possible to detect the exosomes using various methods such as confocal microscopy, fluorescence, and surface plasmon resonance (SPR). Here, we targeted the THP-1 (monocyte cell line) derived exosomes using the bioconjugates that were synthesized, and the binding of the exosomes to the InP/ZnS–Anti-CD63 was confirmed using methods for characterizing size as well as surface plasmon resonance. SPR further showed a signal amplification for the same concentrations of exosomes when compared to just the antibody’s binding to the exosome on their own.

## 2. Materials and Methods

All of the materials were purchased from Sigma Aldrich unless otherwise specified.

### 2.1. Synthesis of InP/ZnS QDs

The method used here is according to a previously published procedure by Tessier et al. [14]. Briefly, 0.45 mmol of indium(III) chloride and 2.2 mmol of zinc(II) chloride are mixed in 5.0 mL of oleylamine and heated to 120 °C under vacuum. After 60 min, it is put under a nitrogen atmosphere and heated to 180 °C. Once this temperature is reached, 1.6 mmol of tris-(diethylamino) phosphine is quickly injected into the mixture. After 20 min of InP core growth, 1 mL of 2.2 M sulfur in trioctylphosphine (TOP-S) is slowly injected over a period of 10 min. At 60 min, the temperature is increased from 180 °C to 200 °C. At 120 min, 1 g of Zn(stearate)_2_ in 4 mL of 1-octadecene (ODE) is slowly injected dropwise over a period of 10 min. The temperature is increased from 200 °C to 220 °C. At 150 min, 0.7 mL of TOP-S is injected slowly over a period of 10 min. The temperature is increased from 220 to 240 °C. At 180 min, 0.5 g of Zn(stearate)_2_ in 2 mL of ODE is slowly added. The temperature is increased from 240 °C to 260 °C. At 210 min, the reaction ends, and the temperature is cooled down to 70 °C and diluted with toluene. The InP/ZnS QDs are then precipitated in ethanol and resuspended in toluene. Further purification was done using a size exclusion column in toluene. This yields-red emitting (~600 nm) InP/ZnS QDs.

### 2.2. Ligand Exchange with MSA and Antibody Conjugation 

The method used below was based on a previous procedure published by Yong et al. [15]. First, 0.30 g of MSA was mixed with 1 mL of toluene and stirred for 10–15 min. Then, 1 mL of QDs (10 mg/mL) were added to the cloudy mixture. This was stirred for 1 min, after which 1 mL of ammonium hydroxide and 1 mL of Milli-Q water were added. This was left to stir overnight. The coloured aqueous layer was purified by precipitating in ethanol and centrifuging. The clear supernatant was discarded, and the pellet was redispersed in 1 mL of Milli-Q water. The water-soluble QDs were stored in the dark at −4 °C. The following antibody was used in this experiment: CD63 Monoclonal antibody (Ts63) from Thermo Fisher Scientific, New Zealand, catalog # 10628D, RRID AB_2532983. Then, 50 µL of 0.05 M of N-hydroxysuccinimide (NHS) and 0.02 M of 1-ethyl-3-(3-dimethylaminopropyl)carbodiimide (EDC) was mixed with 1 mL of 1 mg/mL QD–MSA for 10 mins. Then, 10 µL of a 0.5 mg/mL Anti-CD63 was added to this and further stirred for 2–3 h. The InP/ZnS–Anti-CD63 conjugate was washed using a 30-kDa Amicon centrifugal filter twice with water, after which it was redispersed in 1 mL of water.

### 2.3. Cell Culture

A THP-1 monocyte cell line (ATCC) derived from the peripheral blood of an acute monocytic leukemia patient was grown in Advanced RPMI (Roswell Park Memorial Institute) 1640 cell culture medium (Gibco™, cat. 2633020) containing phenol red and glucose supplemented with 1% (4 mM) of GlutaMAX™ (Gibco™, USA, cat. 35050061) and with reduced 10% fetal bovine serum (FBS) supplementation. THP-1 cells were grown in T75 (Nunc™EasYFlask™,Thermo Fisher, New Zealand cat. 156472) with a seeding density of 2.1 × 10^6^ cells/mL in the condition described above for two days. After this time, the medium was collected, and the cells were refreshed with Dulbecco’s Modified Eagle’s Medium (DMEM) containing 5% heat inactivated fetal bovine serum (FBS) for 24 h, and then bathed in 2% Exosome-Depleted FBS (Gibco™, USA, cat. A2720801) for 12 h to eliminate the risks associated with FBS/FCS background and help ensure optimal and consistent results. After cells reached high density (8.4 x 10^6^ cells/mL), the medium was collected and filtered by using sterile filter unit with MF-Millipore MCE membrane (Millex-GS 0.22 μm, Merck Millipore Ltd., UK, cat. R70A98157) and centrifuged for 5 min at 300 *g* to remove any present cells. Supernatant solution then was collected and re-centrifuged for 10 min at 10,000 *g* to remove any possible apoptotic debris. In order to separate microvesicles, the resulting supernatant was purified using the same 0.22-μm sterile filter unit, and then run through the qEV10 column (Izon Science Ltd. New Zealand) based on size exclusion chromatography (SEC). To quantify the protein amount in the exosome samples, method “Protein A280” was applied on a NanoDrop UV-Vis spectrophotometer.

RAW 264.7 murine “macrophage-like” cells with a mouse monocyte macrophage cell line (obtained from the American Type Culture Collection (ATCC)) were cultured in complete growth Dulbecco’s Modified Eagle’s Medium (DMEM) cell culture medium with 10% heat inactivated fetal bovine serum (FBS) (Sigma, St. Louis, MO, USA) and 1% Penicillin (Gibco™, USA, cat.15140163) with a followed subcultivation ratio of 1:3 in a 5% CO_2_ humidified atmosphere at 37 °C.

### 2.4. Fluorescence Microscopy

THP-1 exosomes were incubated with QD-AntiCD63 for 4 h on ice at RT. RAW 264.7 cells were seeded at 3 × 10^5^ cells/mL in a 35-mm imaging dish with an ibidi Polymer Coverslip Bottom and a four-well silicone insert with four defined cell-free gaps (cat. 80466). THP-1 InP/ZnS–Anti-CD63-labeled exosomes and InP/ZnS-AntiCD63 itself were then added to the seeded cell culture, followed by 1 h of incubation at room temperature (RT). Nuclei were labeled with Hoechst 33342 (Thermo Fisher, New Zealand, cat. H1399) and viewed immediately. Live cell observation was performed on an Olympus IX53 inverted microscope by using excitation/emission wavelength (Ex/Em) = 346/460 nm for the nuclear staining, Ex/Em = 490/525 nm for the QDs labeled THP-1 derived exosomes, and Ex/Em = 596/615 nm for imaging InP/ZnS-AntiCD63.

### 2.5. Transmission Electron Microscopy

The size and crystallinity of the particles were determined using TEM. The analysis was conducted using a 200-KV JEOL 2100F (JEOL, Tokyo, Japan). For the InP/ZnS QDs, 5 µL of sample was pipetted onto carbon-coated 300-mesh copper grids. After 30 min of adsorption, the excess suspension was removed using filter paper, and the sample was left to dry completely overnight.

For THP-1 derived exosomes, the carbon-coated copper grids were plasma-treated for 5 min, and 5 µL of the sample was pipetted on the grid. After 5 min, the excess sample was removed using a filter paper, and 5 µL of 4% uranyl acetate was placed on the grid and left for six min before the grid was washed with ample amounts of DI water. The sizes were measured using Gatan Microscopy Suite Software 3.0.

### 2.6. Nanoparticle Tracking Analysis

For nanoparticle tracking analysis (NTA), the Nanosight NS300 (Malvern Instruments, UK) was used to determine the size measurements of the exosome and InP/ZnS–Anti-CD63 conjugate sample. Analyses were performed on instruments with a 488-nm laser and a syringe pump.

Fourier Transform Infrared Spectroscopy (FTIR):

The FTIR spectra was obtained for all three materials using the Bruker Alpha II spectrometer. Each sample was drop-casted onto the diamond on the attenuated total reflectance unit and left to dry, after which a spectra was recorded. A background measurement using the respective solvent systems was also done.

### 2.7. Dynamic Light Scattering

The hydrodynamic diameter and zeta potentials were measured for the particles using the Zetasizer ZS90 (Malvern Instruments, UK). Each reading reported was an average of 12 measurements.

### 2.8. Surface plasmon resonance (SPR)

SPR measurements were conducted on the Biacore X100 with HBS-EP buffer (GE Healthcare, Life sciences, Denmark) as the running buffer. The flow rate that was used for the measurements was 5 µL/min. The following antibody was used in this experiment: Goat anti-Mouse IgG1 Cross-Adsorbed Secondary Antibody from Thermo Fisher Scientific, New Zealand, catalog #A10538, RRID AB_2534038. The secondary antibody was immobilized onto a CM3 chip using 0.4-M 1-ethyl-3-(3-dimethylaminopropylcarbodiimide) and 0.1 M of NHS in water to get an immobilization level of 2000 response units (RU). Then, a mix of InP/ZnS–Anti-CD63 conjugates (at the same concentration) and exosomes (at varying protein concentrations) was run through the system for 18 min. The same was done for mixes of antibody (at 5 µg/mL) and exosomes (at varying protein concentrations). The response from the blank solution of InP/ZnS–Anti-CD63 or just antibody with no exosomes was subtracted from the subsequent responses to get responses from the change in exosome concentration (shown in Figures S3 and S4). Through the experiments, one of the flow cells on the chip was used as a reference, and was kept blank to account for refractive index changes from the different buffer systems.

### 2.9. Photoluminescence Spectroscopy 

Steady-state photoluminescence measurements on InP/ZnS QDs and exosome conjugates in the range of 520–800 nm were acquired using the FLS-980 Photoluminescence spectrometer. Quantum yields were calculated using the integrating sphere. The samples were excited at 480 nm.

## 3. Results

### 3.1. Synthesis and Characterization of the InP/ZnS QDs

The synthesis of the InP/ZnS QDs was done using oleylamine as a coordinating solvent and the precursor tris (diethylamino) phosphine as a scalable and economical protocol [14]. Oleylamine has a high boiling point that makes it suitable for use in high-temperature syntheses, resulting in InP/ZnS QDs that are soluble in organic solvents such as toluene, with quantum yields of about 37.40%.

A further ligand exchange was done to render the QDs hydrophilic, and therefore better suited to aqueous environments, such as an extracellular matrix. The QDs were functionalized with MSA, which has a carboxylic acid functionality. This was done using a basic solution of ammonia, which was left stirring with the QDs in toluene and the new ligand MSA over a period of 12 h. The basic solution can deprotonate the thiol group on the MSA, and encourage the reaction of this with the ZnS surface on the QDs.

**Figure 1:**
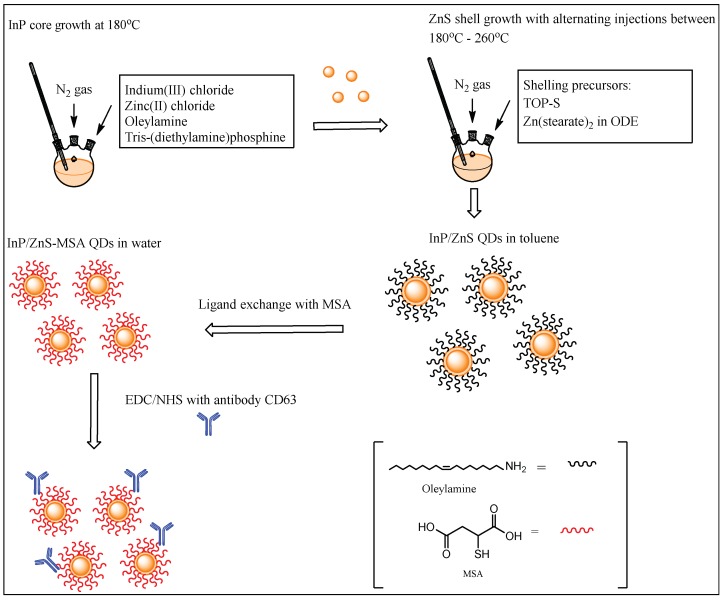
Synthetic scheme for the InP/ZnS quantum dots (QDs) conjugated to the antibody anti-CD63.

The amine functional groups in biomolecules such as antibodies allow for an amide covalent bond to form via a carbodiimide linkage. The synthetic scheme shown in Figure 1 shows the steps for the synthesis of the QDs along with their subsequent functionalization to achieve fluorescent and specific QD conjugates.

**Figure 2:**
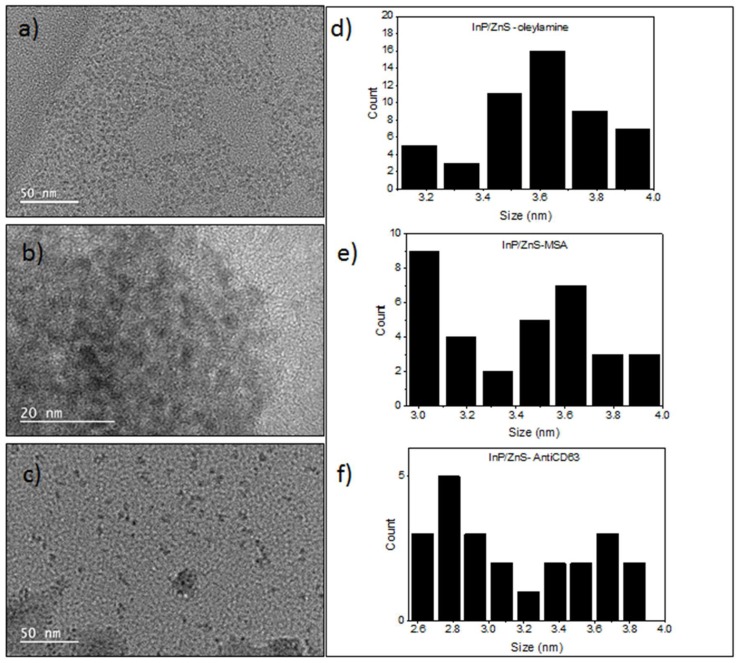
(**a**) TEM and (**b**) size distribution of InP/ZnS QDs dispersed in toluene. (**c**) TEM and (**d**) size distribution of InP/ZnS QDs dispersed in water. (**e**) TEM and (**f**) size distribution of InP/ZnS–Anti-CD63 conjugate.

The TEM images in Figure 2 show the InP/ZnS QDs with the oleylamine ligand and after ligand exchange both exhibiting an average size of around 3.6 nm. The water-soluble QDs showed little aggregation in the images, but stayed in solution for weeks after their synthesis. There is a visible reduction in size with the InP/ZnS–Anti-CD63 conjugates, which was observed from the TEM images from an average size of 3.6 nm to between 2.6–3.8 nm for the InP/ZnS–AntiCD63 conjugates. The size difference could be due to the ligand exchange. InP/ZnS QDs that have carbon-based oleylamine ligands and MSA ligands on them are harder to image on TEM due to their lower contrast on carbon-based grids. Limited aggregation was seen from the InP/ZnS–Anti-CD63 conjugates as well.

Table 1 shows a summary of the different characteristics of the QDs that we synthesized and compares them to similarly prepared InP/ZnS QDs. The quantum yields are visibly higher for the InP/ZnS–Anti-CD63 conjugate and the original oleylamine-coated QDs. There were shifts in emission maximums after changes in the surface chemistry from an original 600 nm to 590 nm for the QD–MSA and 580 nm for the InP/ZnS–Anti-CD63 (Figure S1). Furthermore, the full-width half-maximum (FWHM) also shows changes with the different surface chemistries. FWHM is an indication of size distribution of the photoluminescent sample and becomes broader with the ligand exchange and the conjugation of the antibody indicating a more polydisperse sample, which could be from the unconjugated QD.

The ligand exchange was confirmed from the easy dispersibility of the QDs in water as opposed to toluene. DLS was also used to show a smaller hydrodynamic diameter of the QDs at 4.27 nm, and a large negative zeta potential value of −37.8 mV indicated a stable dispersion in aqueous systems (shown in Table 1). Further to this, a significant reduction in quantum yield was observed for the water-soluble QDs, which is typical of ligand exchanges [16], and can be attributed to the use of ammonia, which can etch the QDs. 

Antibodies are large proteins that specifically bind to targets, such as for example, proteins on the surface of a cell membrane or extracellular vesicle. They have been used for biosensing, as they provide specificity for the respective sensors [3,17]. The synthesis of the InP/ZnS–Anti-CD63 conjugate was confirmed using DLS. An increase in size from the original 4.27 nm to 86.30 nm for the InP/ZnS–Anti-CD63 conjugate was observed, which comes from the attachment of a large protein to the QD. Further to this, zeta potential values can give a good indication about the change in surface chemistries [18,19]. This value also changed from the original −37.80 mV for the QD–MSA to −7.53 mV for the InP/ZnS–Anti-CD63 conjugate.

**Figure 3:**
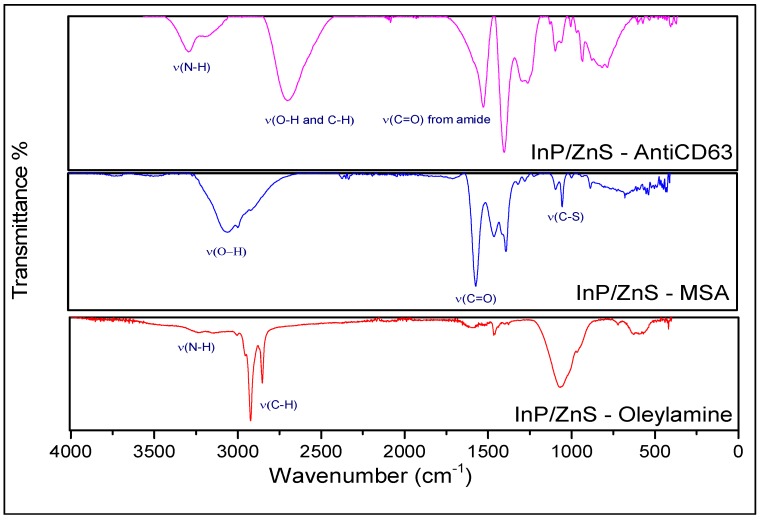
Fourier transform infrared spectroscopy (FTIR) spectra of the InP/ZnS–oleylamine, InP/ZnS–MSA, and InP/ZnS–Anti-CD63.

FTIR (Figure 3) of the as-prepared QDs (InP/ZnS–oleylamine), MSA QDs (InP/ZnS–MSA) and the QD–Antibody (InP/ZnS–Anti-CD63) confirmed the ligand exchange, as it shows a C=O stretch at 1700 cm^−1^ and an OH stretch at 3010 cm^−1^. Further to this, the sharp C–H stretch at 2900 cm^−1^ is not prominent in the FTIR spectra of InP/ZnS–MSA.

The FTIR of InP/ZnS–Anti-CD63 also showed characteristic amide peaks (1650 cm^−1^, 1540 cm^−1^) from the antibody. The stretches at 3300 cm^−1^ are from a primary amine group possibly on the antibody. The strong peak at 1650 cm^−1^ is due to a C=O stretch within an amide functional group, which is also characteristic of proteins (see Figure S2). The peak at 1510 cm^−1^ is due to the N–H in-plane bend from an amide group.

### 3.2. Testing of the InP/ZnS–Anti-CD63 Conjugate

**Figure 4:**
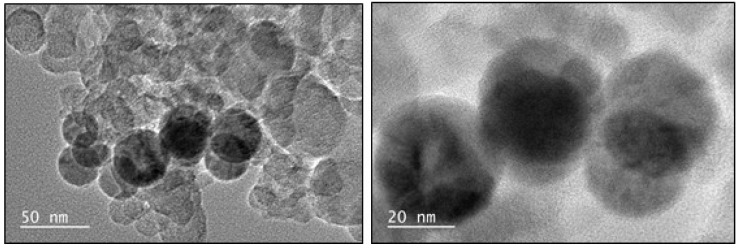
TEM image of THP-1 derived exosomes.

The exosomes used for this work were derived from THP-1 human monocytes, which were collected and purified using optimized procedures (see methods, cell culture). THP-1 cells are leukemia-derived and are known to produce more exosomes (as in most cancer cell lines) [22]. Firstly, the collected media (free of serum-derived exosomes) were immediately centrifuged to remove cellular debris, which could contaminate the sample. This was then followed by filtration and subsequent size exclusion column, which is one of the favorable methods of purification, as it results in high yields and purity. The exosomes were imaged on TEM (Figure 4, which verified the size and morphology to be about 50 nm. 

Conjugation of the InP/ZnS–Anti-CD63 to the exosomes was also confirmed using NTA, which showed an increase in size from 96.6 nm of the THP-1 derived exosomes to 129.5 nm for the mixed solution of InP/ZnS–Anti-CD63 + exosome (Table 2). According to TEM, the size of an exosome is around 50 nm to 100 nm. An exosome with a diameter of 47 nm, for example, on attachment to the InP/ZnS–Anti-CD63 (of sizes around 3.0 nm) would lead to an increase in size to 50 nm. A similar increase in hydrodynamic diameter was seen using dynamic light scattering. In this case, the diameters that are reported using DLS are therefore more accurate and closer to what is expected than the nanosight. However, as is visible from the TEM images, the THP-1-derived exosome sample was quite polydisperse, with sizes ranging between 50 nm to a 100 nm, which results in a discrepancy in the measurement across the two methods that were used to determine hydrodynamic diameter. Hydrodynamic diameter increases also do not fully confirm attachment of the exosome to the conjugate.

To verify the binding of InP/ZnS–Anti-CD63 with exosomes, surface plasmon resonance on the Biacore X100 was used. A secondary anti-mouse antibody was immobilized to the carboxymethyl dextran matrix on the CM3 gold chip using the same amide coupling method. The immobilization was done on one flow cell. The second flow cell was kept blank to allow for reference subtraction. The secondary anti-mouse antibody was able to capture the primary antibody (anti-CD63) to give a response of up to 200 RU. For the InP/ZnS–Anti-CD63 conjugate, this primary binding response was at 1800 RU, which can be attributed to the signal amplification that the QD caused. This was subsequently used for the capture of the THP-1-derived exosomes. The concentration of the antibody and the InP/ZnS–Anti-CD63 was kept constant, and the exosome protein concentrations used were 0.01 mg/mL, 0.1 mg/mL, 0.5 mg/mL, and 1 mg/mL. The response of the primary antibody and the InP/ZnS–Anti-CD63 conjugates by themselves were subtracted from all of these concentrations to give the response from the exosomes.

**Figure 5:**
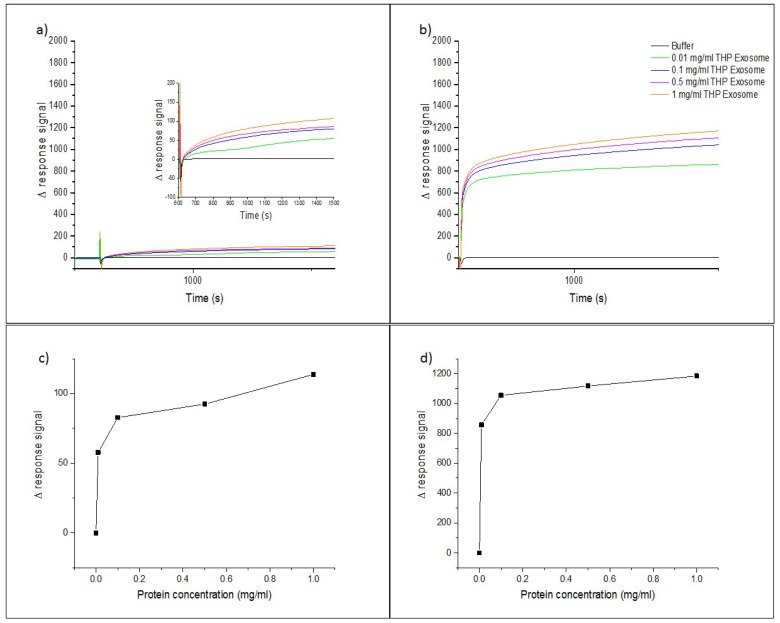
Surface plasmon measurements of the THP-1 derived exosomes using just the antibody (**a**) and the InP/ZnS–Anti-CD63 (**b**). Binding curves of the response of the sensor chip to the binding of the exosomes using the Anti-CD63 (**c**) and InP/ZnS–Anti-CD63 (**d**). For the InP/ZnS–Anti-CD63, the binding shows a steep increase for the lowest protein concentration of 0.01 mg/mL. The higher protein concentrations then increase gradually.

The SPR measurements seen in Figure 5 confirm a number of things. The measurements of just the antibody and the exosome confirm that the anti-CD63 does target a membrane protein on the THP-1-derived exosomes, and gives the response from the population of the exosomes that contain this protein. Further to this, the measurements confirm the presence of the bioconjugate due to the amplified response from the bioconjugate. Additionally, they confirm the binding of the QD conjugate to the exosome with an amplified response for all of the concentrations.

Figure 5c,d further shows the relationship between the binding response to each of the varied protein concentrations that were measured. This showed a gradual but steep increase from the blank to the exosome sample with 0.01 mg/mL of protein. This increase was steeper with the InP/ZnS–Anti-CD63 and the higher protein concentrations. Further to this, the response increased very slowly after the lowest protein concentration tested.

**Figure 6:**
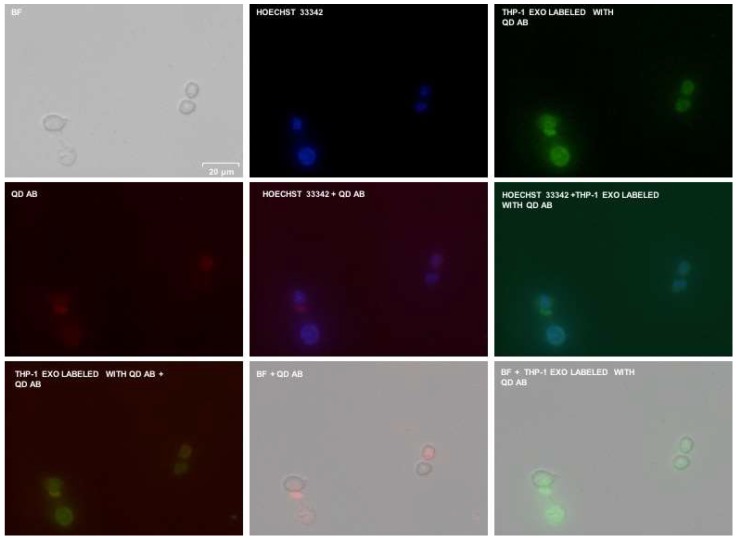
Fluorescence microscopy performed on RAW 264.7 with InP/ZnS–Anti-CD63 labeled exosomes (THP-1 derived) and InP/ZnS–Anti-CD63.

Fluorescence microscopy (Figure 6) also shows the successful labeling of cells and exosomes with InP/ZnS–Anti-CD63, which corresponded to laser excitation at 490 nm and a 525-nm emission filter. A InP/ZnS–Anti-CD63 concentration of 1 mg/mL was used for THP-1 derived exosomes labeling, but with the protein ratio of 1 (exosomes):3 (InP/ZnS–Anti-CD63).

## 4. Discussion

The suite of techniques that is used for the detection and analysis of exosomes has several issues, ranging from size detection limits to complicated instrumentation. Exosomes have further been implicated in several diseases such as Parkinson’s [23], cancer [24], and cardiovascular diseases [25]. Therefore, the detection of these biomarkers will be useful a method for the detection of the above diseases. Techniques such as SPR have made it possible to detect and analyze a sample of exosomes [9]. Further to this, SPR has also been used for the detection of clinically relevant exosomes, or exosomes that have a cancer marker on their membrane [26]. However, the preferred method for the isolation of exosomes usually involves the use of size columns, which can significantly dilute the sample. Here, we showed that through the use of a QD conjugated to an antibody, it is possible to amplify the SPR signal that is achieved. This was compared to the response achieved using the same concentration of antibody within the InP/ZnS–Anti-CD63 and the antibody on its own. For the same exosome concentrations, the response using a captured InP/ZnS–Anti-CD63 is much higher than the response from using just the antibody itself. The use of InP/ZnS–Anti-CD63 conjugates for the purposes of signal amplification has been observed for tumor markers using SPR, as well with the further use of a gold nanoparticle for dual signal amplification, which led to a 50-fold increase [27]. The specificity and size of this sensor could be made better and smaller respectively through the use aptamers instead of antibodies, which are easier to work with for in vitro studies. Further to this, methods for isolating the InP/ZnS–Anti-CD63 conjugates could also improve this study, wherein there are no non-specific, non-conjugated QDs in solution.

In addition to SPR, other ways to detect the exosomes using the bioconjugate include the use of confocal microscopy, wherein tracking of the exosome in vivo or in vitro is beneficial for understanding their involvement in processes [28]. The use of InP/ZnS QDs as opposed to cadmium provide a more stable and safer alternative. Their size is small enough to be able to localize accurately. Further improvements to quantum yields for these QDs are still underway. The tuning of their synthesis near-infrared fluorescence would further allow for these nanomaterials to be used in vitro for deep tissue imaging with little background fluorescence interferences [28]. 

In conclusion, non-toxic InP/ZnS QDs were synthesized, ligand-exchanged, and conjugated to an antibody that is specific to exosomes. These materials were characterized using TEM, fluorescence spectroscopy, absorbance spectroscopy, FTIR, and DLS. FTIR and DLS confirmed the ligand exchange, and the latter confirmed the conjugation of the QD to the antibody. The activity of this conjugate was tested against different concentrations of the exosomes using SPR techniques, and was compared to the activity of just the antibody on its own to show that the QD allows for significant signal amplification, which would be beneficial for dilute samples of exosomes.

## Figures and Tables

**Table 1:** Summary table of the physical properties of the QDs. DLS: dynamic light scattering, MSA: mercaptosuccinic acid, PL QY: photoluminescence quantum yield.

QD Conjugates	Zeta Potential (mV)	Hydrodynamic Diameter (nm) or Size	PL QY (%)	Emission Maximum (nm)	PL FWHM (Full-Width Half-Maximum)	Reference
InP/ZnS–oleylamine	-	4.96 (DLS)	37.40%	600	53.00	This work
InP/ZnS–MSA	−37.8	4.27 (DLS)	17.82%	590	55.38	
InP/ZnS–MSA–Anti-CD63	−7.53	86.30 (DLS)	13.83%	580	70.00	
InP/ZnS–MPA (mercaptopropionic acid)	−26.0 ± 9	11.3 ± 0.6 (DLS)				[5]
InP/ZnS in toluene		5 ± 0.5 (TEM)		634		[20]
InP/ZnS (with PEGylated phospholipids)	−8.63	58.50 (DLS)				
InP/ZnS in hexane		4.9–12.6 (DLS)	15%	555	62.00	[21]
InP/ZnS–folic acid		17–24 (DLS)				

**Table 2:** Size results for the THP-1 derived exosomes and a mix of InP/ZnS–Anti-CD63 + THP-1 derived exosomes. The mix was incubated together in a fridge for an hour.

Sample	THP-1 Derived Exosomes	InP/ZnS–Anti-CD63 + THP-1 Derived Exosomes
Hydrodynamic diameter (Nanosight)	96.6 ± 7.9 nm	129.5 ± 20.2 nm
Hydrodynamic diameter (DLS)	36.85 nm	52.24 nm
Size (TEM)	50–100 nm	-

## Supplementary Materials

The following are available online at http://www.mdpi.com/1424-8220/18/10/3308/s1, Figure S1: Absorption and fluorescence of the InP/ZnS-Oleylamine, InP/ZnS-MSA and InP/ZnS-AntiCD63. Figure S2: FTIR spectra of Oleylamine, MSA and Anti-CD63. Figure S3: Immobilisation of the secondary antibody to the CM3 chip. Figure S4: Response after injection of the THP-1 derived exosomes onto the secondary antibody.

## References

[1] Medintz I.L., Uyeda H.T., Goldman E.R., Mattoussi H. (2005). Quantum dot bioconjugates for imaging, labelling and sensing. Nat. Mater..

[2] Boriachek K., Islam M.N., Gopalan V., Lam A.K., Nguyen N.-T., Shiddiky M.J.A. (2017). Quantum dot-based sensitive detection of disease specific exosome in serum. Analyst.

[3] Han H.-S., Niemeyer E., Huang Y., Kamoun W.S., Martin J.D., Bhaumik J., Chen Y., Roberge S., Cui J., Martin M.R. (2015). Quantum dot/antibody conjugates for in vivo cytometric imaging in mice. Proc. Natl. Acad. Sci. USA.

[4] Zrazhevskiy P., Gao X. (2013). Quantum dot imaging platform for single-cell molecular profiling. Nat. Commun..

[5] Brunetti V., Chibli H., Fiammengo R., Galeone A., Malvindi M.A., Vecchio G., Cingolani R., Nadeau J.L., Pompa P.P. (2013). InP/ZnS as a safer alternative to CdSe/ZnS core/shell quantum dots: In vitro and in vivo toxicity assessment. Nanoscale.

[6] Boriachek K., Islam M.N., Möller A., Salomon C., Nguyen N.T., Hossain M.S.A., Yamauchi Y., Shiddiky M.J.A. (2018). Biological Functions and Current Advances in Isolation and Detection Strategies for Exosome Nanovesicles. Small.

[7] Lobb R.J., van Amerongen R., Wiegmans A., Ham S., Larsen J.E., Möller A. (2017). Exosomes derived from mesenchymal non-small cell lung cancer cells promote chemoresistance. Int. J. Cancer.

[8] Andreu Z. (2014). Tetraspanins in extracellular vesicle formation and function. Front. Immunol..

[9] Rupert D.L.M., Lässer C., Eldh M., Block S., Zhdanov V.P., Lotvall J.O., Bally M., Höök F. (2014). Determination of Exosome Concentration in Solution Using Surface Plasmon Resonance Spectroscopy. Anal. Chem..

[10] Willms E., Johansson H.J., Mäger I., Lee Y., Blomberg K.E.M., Sadik M., Alaarg A., Smith C.I.E., Lehtiö J., EL Andaloussi S. (2016). Cells release subpopulations of exosomes with distinct molecular and biological properties. Sci. Rep..

[11] Van Der Pol E., Hoekstra A.G., Sturk A., Otto C., Van Leeuwen T.G., Nieuwland R. (2010). Optical and non-optical methods for detection and characterization of microparticles and exosomes. J. Thromb. Haemost..

[12] Van der Pol E., Coumans F.A.W., Grootemaat A.E., Gardiner C., Sargent I.L., Harrison P., Sturk A., van Leeuwen T.G., Nieuwland R. (2014). Particle size distribution of exosomes and microvesicles determined by transmission electron microscopy, flow cytometry, nanoparticle tracking analysis, and resistive pulse sensing. J. Thromb. Haemost..

[13] Dragovic R.A., Gardiner C., Brooks A.S., Tannetta D.S., Ferguson D.J.P., Hole P., Carr B., Redman C.W.G., Harris A.L., Dobson P.J. (2011). Sizing and phenotyping of cellular vesicles using Nanoparticle Tracking Analysis. Nanomedicine.

[14] Tessier M.D., Dupont D., De Nolf K., De Roo J., Hens Z. (2015). Economic and Size-tunable Synthesis of InP/ZnE (E = S,Se) Colloidal Quantum Dots. Chem. Mater..

[15] Yong K.T., Ding H., Roy I., Law W.C., Bergey E.J., Maitra A., Prasad P.N. (2009). Imaging pancreatic cancer using bioconjugated inp quantum dots. ACS Nano.

[16] Nann T. (2005). Phase-transfer of CdSe@ZnS quantum dots using amphiphilic hyperbranched polyethylenimine. Chem. Commun..

[17] Trilling A.K., Beekwilder J., Zuilhof H. (2013). Antibody orientation on biosensor surfaces: A minireview. Analyst.

[18] Korpany K.V., Mottillo C., Bachelder J., Cross S.N., Dong P., Trudel S., Friščić T., Blum A.S. (2016). One-step ligand exchange and switching from hydrophobic to water-stable hydrophilic superparamagnetic iron oxide nanoparticles by mechanochemical milling. Chem. Commun..

[19] Liu Y., Purich D.L., Wu C., Wu Y., Chen T., Cui C., Zhang L., Cansiz S., Hou W., Wang Y. (2015). Ionic Functionalization of Hydrophobic Colloidal Nanoparticles To Form Ionic Nanoparticles with Enzymelike Properties. J. Am. Chem. Soc..

[20] Liu J., Hu R., Liu J., Zhang B., Wang Y., Liu X., Law W.-C., Liu L., Ye L., Yong K.-T. (2015). Cytotoxicity assessment of functionalized CdSe, CdTe and InP quantum dots in two human cancer cell models. Mater. Sci. Eng. C.

[21] Bharali D.J., Lucey D.W., Jayakumar H., Pudavar H.E., Prasad P.N. (2005). Folate-Receptor-Mediated Delivery of InP Quantum Dots for Bioimaging Using Confocal and Two-Photon Microscopy. J. Am. Chem. Soc..

[22] Saha B., Momen-Heravi F., Kodys K., Szabo G. (2015). MicroRNA Cargo of Extracellular Vesicles from Alcohol-Exposed Monocytes Signals Naïve Monocytes to Differentiate into M2 Macrophages. J. Biol. Chem..

[23] Wu X., Zheng T., Zhang B. (2017). Exosomes in Parkinson’s Disease. Neurosci. Bull..

[24] Sun W., Luo J., Jiang H., Duan D.D. (2018). Tumor exosomes: A double-edged sword in cancer therapy. Acta Pharmacol. Sin..

[25] Osada-Oka M., Shiota M., Izumi Y., Nishiyama M., Tanaka M., Yamaguchi T., Sakurai E., Miura K., Iwao H. (2017). Macrophage-derived exosomes induce inflammatory factors in endothelial cells under hypertensive conditions. Hypertens. Res..

[26] Sina A.A.I., Vaidyanathan R., Dey S., Carrascosa L.G., Shiddiky M.J.A., Trau M. (2016). Real time and label free profiling of clinically relevant exosomes. Sci. Rep..

[27] Wang H., Wang X., Wang J., Fu W., Yao C. (2016). A SPR biosensor based on signal amplification using antibody-QD conjugates for quantitative determination of multiple tumor markers. Sci. Rep..

[28] Shen L.-M., Quan L., Liu J. (2018). Tracking Exosomes in Vitro and in Vivo To Elucidate Their Physiological Functions: Implications for Diagnostic and Therapeutic Nanocarriers. ACS Appl. Nano Mater..

