# Supplementary material for: Cadmium-Free Quantum Dots as Fluorescent Labels for Exosomes
Source: Sensors (Basel). 2018 Oct 2;18(10):3308. doi: 10.3390/s18103308 (PMC6210340; doi:10.3390/s18103308)
Supplement: Supplementary file 1 [file sensors-18-03308-s001.pdf]

Supporting Information

Cadmium-Free Quantum Dots as Fluorescent Labels for Exosomes

Garima Dobhal, Deanna Ayupova, Geoffry Laufersky, Zeineb Ayed, Renee V. Goreham and Thomas Nann

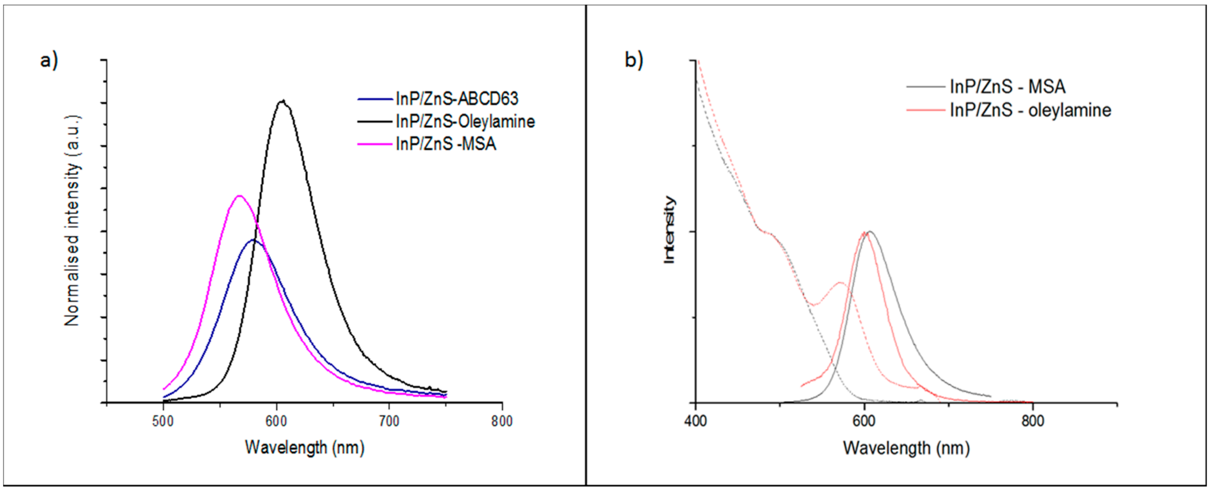

Figure S1. a) Fluorescence intensity and emission maximum changes with ligand exchange and conjugation of an antibody. Each fluorescence spectra was normalized to the absorbance at the excitation wavelength of 480 nm. b) Absorption and emission characteristics of the InP/ZnS-oleylamine and InP/ZnS-MSA.

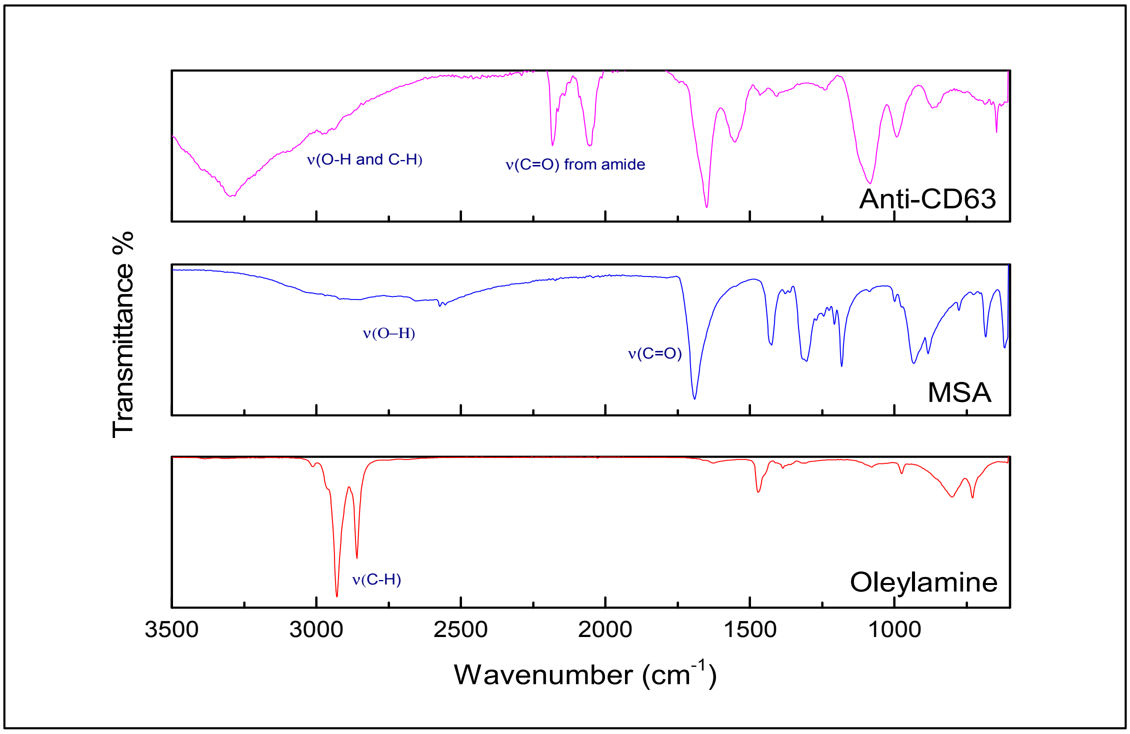

Figure S2. FTIR spectra of oleylamine, MSA and anti-CD63. There is a shift in the amide peaks of the antibody when compared to the FTIR of the InP/ZnS-AntiCD63. For the InP/ZnS-MSA, a similar change is seen for the peak at 1700 cm^{-1}.

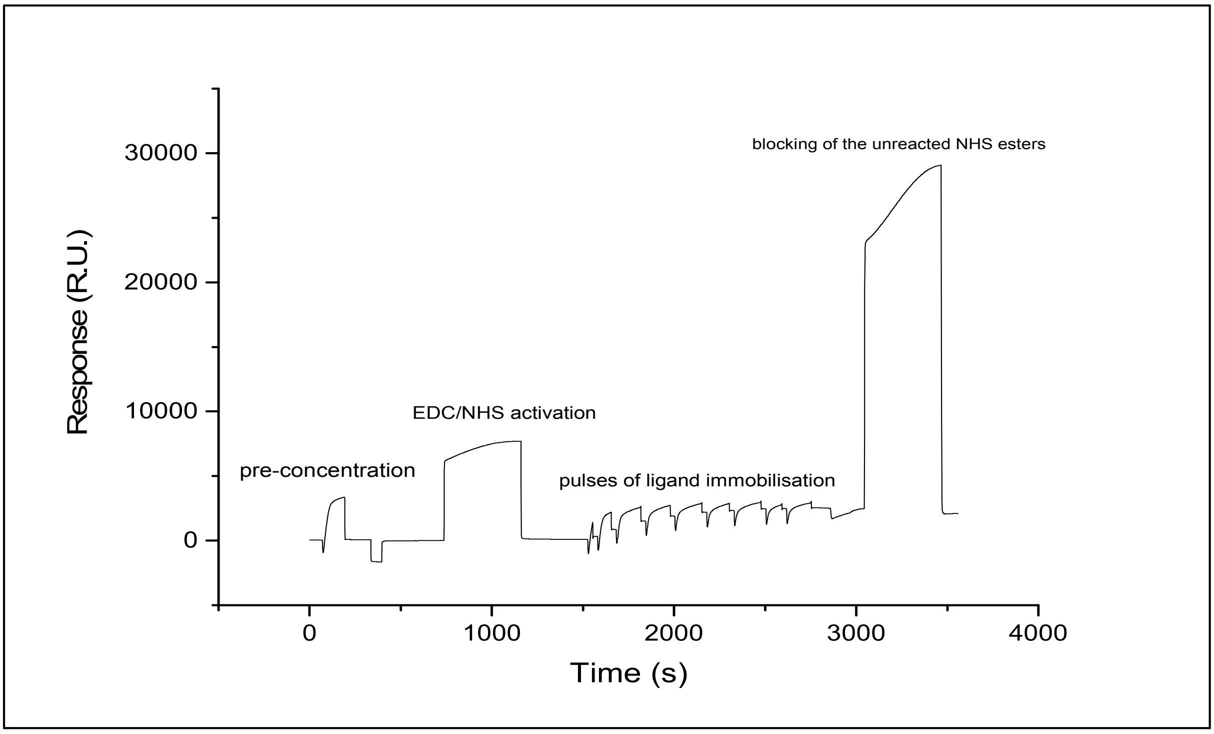

Figure S3: Immobilisation of the secondary antibody onto chip CM3.

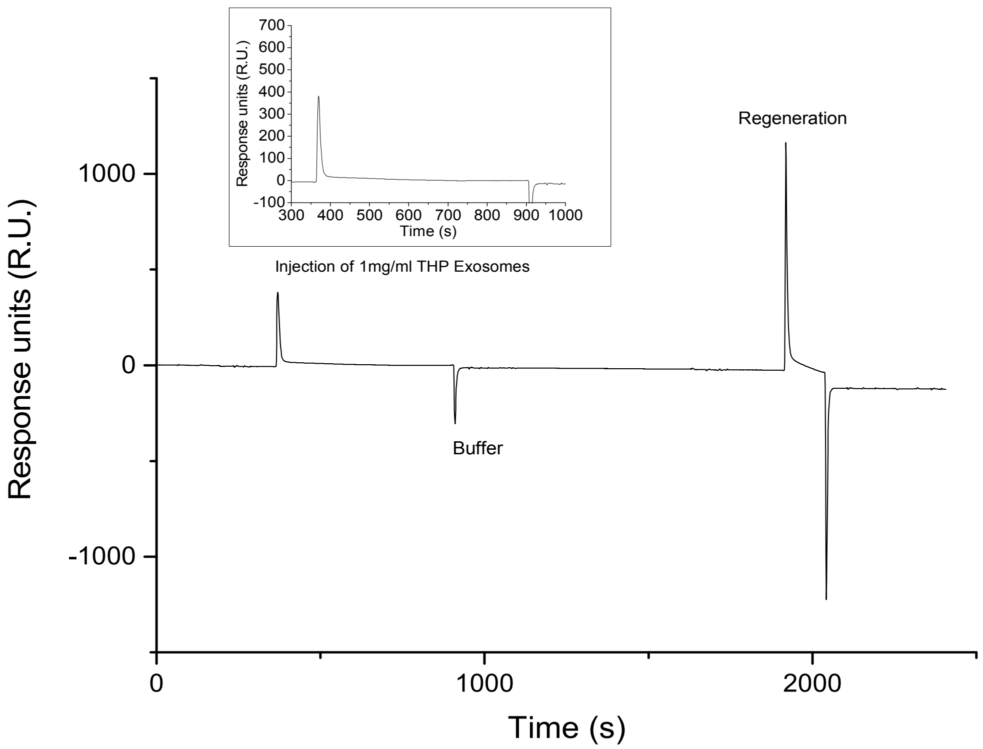

Figure S4. Injection of just exosomes on the secondary antibody gives a very little response indicating the non-specificity of the antibody to the exosomes themselves. Inset shows close-up of the injection time and response.
